# Indoleamine 2,3-Dioxygenase: Expressing Cells in Inflammatory Bowel Disease—A Cross-Sectional Study

**DOI:** 10.1155/2013/278035

**Published:** 2013-10-27

**Authors:** Janette Furuzawa-Carballeda, Gabriela Fonseca-Camarillo, Guadalupe Lima, Jesús K. Yamamoto-Furusho

**Affiliations:** ^1^Department of Immunology and Rheumatology, Instituto Nacional de Ciencias Médicas y Nutrición Salvador Zubirán, Vasco de Quiroga No. 15, Colonia Sección XVI, Tlalpan, 14000 Mexico City, DF, Mexico; ^2^Department of Gastroenterology, Inflammatory Bowel Disease Clinic, Instituto Nacional de Ciencias Médicas y Nutrición Salvador Zubirán, Vasco de Quiroga No. 15, Colonia Sección XVI, Tlalpan, 14000 Mexico City, DF, Mexico

## Abstract

*Aim*. To characterise and enumerate IDO^+^ cells, Tregs, and T cell subsets in patients with ulcerative colitis (UC) and Crohn's disease (CD) with regard to their clinical activity. *Methods*. Ten active UC (aUC), 10 inactive UC (iUC), 6 aCD, and 8 iCD patients and 10 healthy individuals were included in the study. Circulating Foxp3-, IDO-, IL-17A-, IL-4-, IFN-**γ**-, and IL-10-expressing CD4^+^ T cells were quantitated by flow cytometry. Interleukin-17-expressing cells, CD25^+^/Foxp3^+^ Tregs, and CD123^+^/IDO^+^ plasmacytoid dendritic cells were evaluated in intestinal biopsies from 10 aUC, 6 aCD, and 10 noninflamed tissues. *Results*. All CD4^+^ T subsets were increased in aIBD patients compared with healthy donors. Meanwhile, frequency of CD8**α** ^+^/CD16^+^/IDO^+^, CD8**α** ^+^/CD56^+^/IDO^+^, CD8**α** ^+^/CD80^+^/IDO^+^, CD8**α** ^+^/CD123^+^/IDO^+^ large granular nonlymphoid cells, and CCR6^+^/CD123^+^/IDO^+^ plasmacytoid dendritic cells was higher in aIBD patients versus healthy donors or iIBD patients. Tissue IL-17A^+^ cells were present in higher amounts in aIBD versus noninflamed controls. IDO- and Foxp3-expressing cells were increased in aUC versus aCD patients and noninflamed tissues. *Conclusions*. The findings represent an original work in Mexican Mestizo patients with IBD. It shows that Tregs and IDO-expressing cells are increased with regard to disease activity. These cells could significantly shape inflammatory bowel disease pathophysiology, severity, and tolerance loss.

## 1. Introduction

Inflammatory bowel disease (IBD) including ulcerative colitis (UC) and Crohn's disease (CD) is a chronic inflammatory disorder of the gastrointestinal tract. Thus far, the aetiology is not completely understood. Furthermore, initiation and exacerbation of the inflammatory process seem to be due to massive mucosal immune response [1]. Therefore, it is not preposterous to correlate the chronic relapsing inflammation with a tolerance dysregulation mechanism or an aberrant immune response to intestinal flora in a context of genetic predisposition [2].

In this vein, the catabolism of tryptophan, by the enzyme indoleamine 2,3-dioxygenase (IDO) expressed in plasmacytoid dendritic cells, generates kynurenines, 3-hydroxyanthranilic, and quinolic acids, molecules with the ability to exert cytotoxic action on T, B, and NK cells, but not on DCs themselves [3]. Moreover, deprivation of tryptophan by IDO halts the proliferation of T cells at mid-G_1_ phase, which in concert with the proapoptotic activity of kynurenine leads to diminishing T cell-mediated immune responses and the subsequent development of immune tolerance [4]. IDO has a selective sensitivity for Th1 over Th2 cells to tryptophan metabolites, suggesting a potential role for Th2 differentiation [4–10]. IDO-competent DCs have shown to induce CD4^+^/CD25^hi^ regulatory T cells (Tregs) *in vivo*, and Treg-expressed glucocorticoid-induced tumour necrosis factor receptor (GITR) which in turn can use IDO^+^ DCs to expand their own population in a positive feedback loop [11, 12].

Thus, quantitative and functional modifications of Tregs and IDO-producing cells might play a role in the pathogenesis and disease activity of autoimmune systemic disorders including UC and CD.

Above and beyond, the role of T cells in the pathogenesis of IBD up until now is not clear. The traditional Th1/Th2 paradigm has not been very helpful to explain several aspects of the illness, and the description of T helper 17 (Th17) cells and their biology opened new possibilities in the study of the pathogenesis of IBD as well as other autoimmune diseases. Th17 cells have been found to be elevated in serum and intestinal tissue from active IBD patients. However, IL-17 has not been detected in inactive IBD tissue [13, 14]. The recent discovery and characterization of Th17 and their signature cytokines (IL-17) represent a hallmark in T cell immunobiology by providing a new, distinctive pathway for communication between adaptive and innate immunity [15].

Thus, the aim of this study was to characterise and to enumerate circulating IDO-producing cells and Foxp3-expressing CD4^+^ T cells as well as IL-17A-, IL-4-, IFN-*γ*-, and IL-10-expressing CD4^+^ T cell subsets by flow cytometry. On the other hand, we determine the number of tissue IL-17A-secreting cells, CD25^+^/Foxp3^+^ cells, and CD123^+^/IDO^+^ plasmacytoid dendritic cells by immunohistochemistry in Mexican Mestizo patients with UC or CD in regard to their clinical activity.

## 2. Material and Methods

### 2.1. Subjects

For this exploratory, observational, and cross-sectional study, 10 active UC (aUC), 10 inactive UC (iUC), 6 active CD (aCD), and 8 inactive CD (iCD) patients were included. UC diagnosis was performed by the presence of the following criteria: a history of diarrhoea or blood in stools, macroscopic appearance by endoscopy, and biopsy compatible with UC. Relevant clinical and demographic information in all UC patients was collected from medical records: gender, age at diagnosis, familial aggregation, smoking history, previous appendectomy, disease evolution, extension, extraintestinal manifestations, medical or surgical treatment, and clinical course of disease. The clinical course of disease was defined as active then inactive (first episode with activity and then long-term remission for more than 5 years); intermittent activity (2 relapses in a year); chronic continual activity (>2 relapses or persistent activity despite of medical conventional therapy) as previously described [16–19].

In addition, 10 healthy donors (HD) age-matched who volunteered were included as controls. HDs were also interviewed in order to discard any known autoimmune disease, use of immunosuppressors and prednisone, and concurrent infections.

A sample of venous blood (10 mL) was obtained from each subject. Peripheral blood mononuclear cells (PBMCs) were isolated by gradient centrifugation on Lymphoprep (Axis-Shield PoC AS, Oslo, Norway).

### 2.2. Flow Cytometry

For Treg, Th17, Th2, Th1, and IL-10-producing cells, PBMCs were labelled with 5 *μ*L of anti-human CD14-FITC-conjugated and anti-CD4-PeCy5-conjugated monoclonal antibodies (BD Biosciences, San Jose, CA) or CD25-FITC-conjugated and anti-CD4-PeCy5-conjugated monoclonal antibodies (BD Biosciences, San Jose, CA) in separated tubes during 20 min at 37°C in the dark. Cells were permeabilised with 200 *μ*L of cytofix/cytoperm solution (BD Biosciences) at 4°C for 20 min. Intracellular staining was performed with an anti-human Foxp3-PE-, IL-17A-PE-, IL-4-PE-, IFN-*γ*-PE-, or anti-human IL-10-labelled monoclonal antibodies (BD Biosciences).

An electronic gate was made for CD14^−^/CD4^+^ cells or CD4^+^/CD25^hi^. Results are expressed as the relative percentage of IL-17A-, IL-4-, IFN-*γ*-, IL-10-, or Foxp3-expressing cells in each gate.

As isotype control, IgG_1_-FITC/IgG_1_-PE/CD45-PeCy5 mouse IgG_1_, k (BD Tritest, BD Biosciences) was used to set the threshold and gates in the cytometer. We ran an unstained (autofluorescence control) and permeabilised PBMCs sample. Autofluorescence control was compared to single stained cell positive controls to confirm that the stained cells were on scale for each parameter. Besides, BD Calibrite 3 beads were used to adjust instrument settings, set fluorescence compensation, and check instrument sensitivity (BD CaliBRITE, BD Biosciences). Fluorescence minus one (FMO) controls were stained in parallel using the panel of antibodies with sequential omission of one antibody, with the exception of the anti-Foxp3 antibody, which was replaced by an isotype control rather than simply omitted.

To determine IDO cell expression, PBMCs were surface labelled with an anti-human CD8*α*-PE and CD16-PECy5, CD8*α*-PE and CD56-PECy5, CD8*α*-PECy5 and CD80-PE, CD8*α*-PE and CD123-PECy5, CD8*α*-PECy5 and CCR6-PE, or CCR6-PE and CD123-PECy5 monoclonal antibodies (BD Biosciences) in separated tubes during 20 min at 37°C in the dark. Cells were permeabilised and stained with a sheep anti-human-IDO (Chemicon, Temecula, CA) and then with FITC-conjugated-rabbit anti-sheep antibody. Cell subset was analysed by flow cytometry. As control of FITC-labelled-rabbit anti-sheep specificity staining, cells were incubated with surface antibodies and FITC-conjugated-rabbit anti-sheep in the absence of sheep anti-human IDO antibody. An electronic gate was made for each and every one of the surface markers employed. Results are expressed as the relative percentage of IDO-expressing cells in each gate. 

Relative percentage of IL-17A-, IL-4-, IFN-*γ*-, IL-10-, and Foxp3-producing cells was obtained from percentage of CD4^+^/CD14^−^ T cells.

Relative percentage of circulating IDO^+^ cells was obtained from percentage of CD8*α* ^+^/CD16^+^, CD8*α* ^+^/CD56^+^, CD8*α* ^+^/CD80^+^, CD8*α* ^+^/CD123^+^, CD8*α* ^+^/CCR6^+^, and CD123^+^/CCR6^+^, respectively.

### 2.3. Tissue Samples

For *in situ* study a total of 10 patients with definitive diagnosis of aUC calculated by the Mayo Score Activity Index [16] and 6 patients with definitive diagnosis of aCD calculated by the Crohn's Disease Activity Index (CDAI) were enrolled in the study [18, 19]. In IBD patients, colonic mucosal biopsies were sampled from the region with the most severe inflammation, evaluated by colonoscopy. The biopsies from non-inflamed tissue were collected from 10 healthy individuals with constipation and bloating undergoing colonoscopy.

### 2.4. Immunohistochemical Detection of IL-17A-Expressing Cells

In order to determine IL-17A-expressing cells, 4 *μ*m thick sections of available formalin-fixed paraffin embedded tissue were placed on positively charged slides. Sections were deparaffinised and rehydrated through a series of xylene and graded alcohols. Endogenous peroxidase was blocked with 3% H_2_O_2_ for 20 min. A 3% normal serum was employed for 30 min as protein blocker. Tissues were incubated for 18 h at 4°C with rabbit polyclonal anti-human IL-17A antibody (Santa Cruz Biotechnology, Santa Cruz, CA, USA) at 10 *μ*g/mL. Binding was detected by incubating sections for 60 min at room temperature with biotinylated goat anti-rabbit IgG antibody (ABC Staining System; Santa Cruz Biotechnology). Slides were incubated with horseradish peroxidase (HRP) streptavidin for 45 min, followed by incubation with the peroxidase substrate 3,3′-diaminobenzidine (DAB) (Sigma, St Louis, MO, USA) for 10 min. The sections were counterstained with haematoxylin, dehydrated with alcohol and xylene, and mounted in resin. Negative control staining was performed with normal human serum diluted 1 : 100, instead of primary antibody. The reactive blank was incubated with phosphate buffer saline-egg albumin (Sigma) instead of the primary antibody. Both controls excluded nonspecific staining or endogenous enzymatic activities. Morphometric evaluation of the immunostained sections was performed in a blinded manner by light microscope. In brief, IL-17A-expressing cells were counted in at least three optical fields from each slide in X320 high-power magnifications. The average values per slide were used for statistical analysis. Results are expressed as the mean ± standard error of the mean (SEM) of cells quantified by the program Image Pro Plus version 5.1.1.

### 2.5. Double-Staining Procedure

To evaluate CD25^+^/Foxp3^+^ regulatory T cells and CD123^+^/IDO^+^ plasmacytoid dendritic cells a simultaneous detection was performed. A second generation of EnVision G*|*2 Doublestain System (Dako, Glostrup, Denmark) was used for the simultaneous detection of two antigens present at low concentrations within one biopsy. The procedure is a sequential double staining where the first antigen was visualized using horseradish peroxidase (HRP)/3′3′-diaminobenzidine (DAB) and the second antigen was visualized using alkaline phosphatase (AP)/Permanent Red. Briefly, incubation of samples with 200 *μ*L of dual endogenous enzyme block was performed for 5 min. This procedure inhibited endogenous AP, peroxidase, and pseudoperoxidase activity present in tissues. After blocking, tissues were incubated with 200 *μ*L of normal serum as negative control, primary rabbit polyclonal anti-IDO IgG antibody, or mouse monoclonal anti-Foxp3 IgG_1_ antibody (Santa Cruz Biotechnology) at 10 *μ*g/mL, for 15 min, at room temperature. The samples were then incubated with 200 *μ*L polymer/HRP reagent for 10 min. This reagent is an HRP conjugated dextran polymer that also carries antibodies to mouse and rabbit immunoglobulins. The reaction was visualized by incubation of 200 *μ*L DAB plus chromogen for 5–15 min. Tissues were incubated with 200 *μ*L Doublestain Block reagent for 3 min and were then incubated with a normal serum as negative control or second primary rabbit polyclonal anti-CD25 IgG antibody (Santa Cruz Biotechnology) or mouse monoclonal anti-CD123 IgG_1_ antibody (Abcam) at 10 *μ*g/mL for 15 min at room temperature. In the next step, 200 mL rabbit/mouse LINK was added for 10 min. This reagent is a dextran polymer carrying antibodies to mouse and rabbit immunoglobulins. Finally, tissues were incubated with 200 *μ*L of polymer/AP reagent for 10 min. Reaction was visualized by incubation with 200 *μ*L Permanent Red chromogen for 5–20 min. Tissues were counterstained with haematoxylin and mounted in aqueous mounting medium. At least two different sections and two fields (X320) were examined for each biopsy. Double positive CD25^+^/Foxp3^+^ regulatory T cells and CD123^+^/IDO^+^-producing plasmacytoid dendritic cells were assessed by estimating positively staining cells and results were reported as the percentage of immunoreactive cells. Results are expressed as the mean ± SEM of cells quantified by the program Image-Pro Plus version 5.1.1.

### 2.6. Ethical Considerations

This work was performed according to the principles expressed in the Declaration of Helsinki. The study was approved by the ethical committee from our institution, and a written informed consent was obtained from all subjects.

### 2.7. Statistics

Sample size has not been determined for this study; it was merely observational. Descriptive statistics were performed, and categorical variables were compared using the *χ* ^2^ test or Fisher's exact test. One way analysis of variance on ranks by Holm-Sidak Method was performed for all pairwise multiple comparison procedures. Statistical analysis was done using the Sigma Stat 11.2 program (Aspire Software International, Leesburg, VA, USA). Data were expressed as the median, range, and mean ± standard deviation (SD)/standard Error of the mean (SEM). The *P* values smaller than or equal to 0.05 were considered as significant. This study conforms to STROBE statement along with references to STROBE and the broader EQUATOR guidelines [20].

## 3. Results

### 3.1. Demographic Features

We studied a total of 44 individuals who were divided in five groups: aUC (*n* = 10); iUC (*n* = 10); aCD (*n* = 6); iCD (*n* = 8); healthy individuals (*n* = 10). The demographic and clinical variables of patients are summarized in Table 1. Laboratory data are condensed in Table 2. There was a statistical difference in ESR between active IBD (CD and UC) patients and inactive IBD patients (*P* < 0.05; Table 2). aCD had higher levels of CRP versus iCD (*P* < 0.05; Table 2). Haemoglobin concentration in aCD patients was decreased compared with inactive CD patients (*P* < 0.05; Table 2).

### 3.2. Circulating IL-17A-Producing CD4^+^/CD14^−^ T Cells in Patients with UC and CD

**Figure 1:**
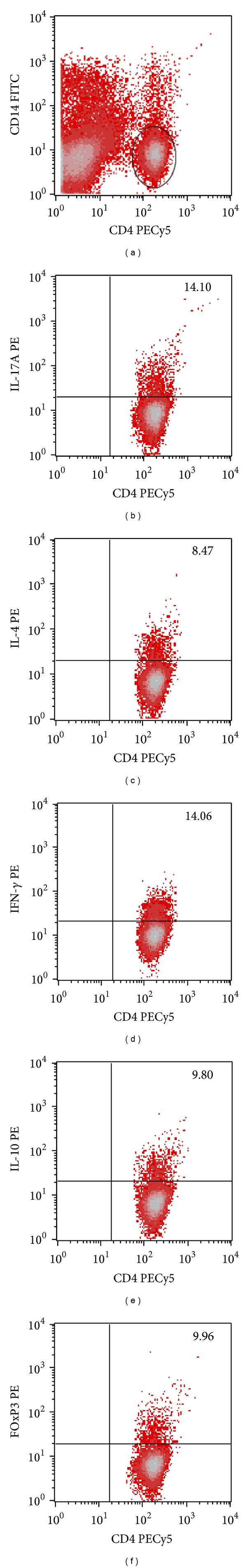
Density plots of cytokine-expressing CD4^+^ T peripheral cells in IBD (UC and CD) patients. (a) CD14^−^/CD4^+^ T cells were determined. (b) From the latter CD14^−^/CD4^+^/IL-17A^+^ cells were defined. (c) From the gate *A* CD14^−^/CD4^+^/IL-4^+^ cells were determined. (d) From the gate *A* CD14^−^/CD4^+^/IFN-*γ* ^+^ cells were defined. (e) From the gate *A* CD14^−^/CD4^+^/IL-10^+^ cells were determined. (f) CD4^+^/CD25^hi^/Foxp3^+^ cells were defined. A total of 50,000–100,000 events were recorded for each sample before any gate setting and analysed with the CellQuest Pro software (BD Biosciences).

**Figure 2:**
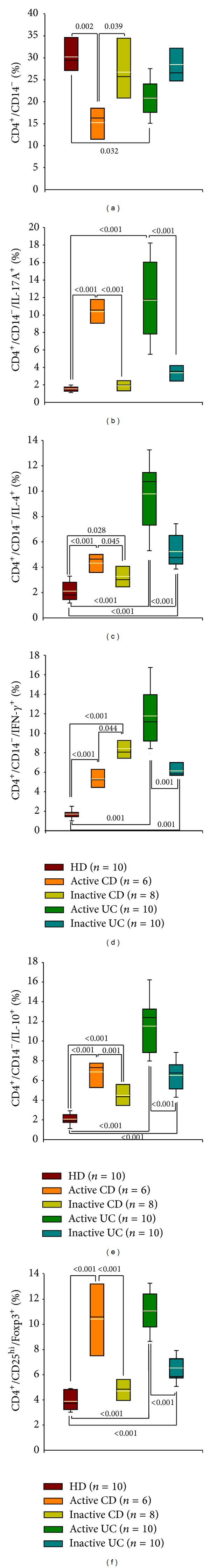
Percentage of cytokine-expressing CD4^+^ T peripheral cells in IBD (UC and CD) patients. Bar graphs show percentage of (a) CD14^−^/CD4^+^ T cells; (b) CD14^−^/CD4^+^/IL-17A^+^; (c) CD14^−^/CD4^+^/IL-4^+^; (d) CD14^−^/CD4^+^/IFN-*γ* ^+^; (e) CD14^−^/CD4^+^/IL-10^+^; (f) CD4^+^/CD25^hi^/Foxp3^+^ cells. Results are expressed as median (black line), 10th, 25th, 75th, and 90th percentiles. Yellow line represents the mean.

Proinflammatory IL-17A-expressing CD4^+^ T cell subset was present in higher amounts in aCD patients versus iCD patients or healthy donors (10.4 ± 0.6% versus 2.0 ± 0.2% or 1.5 ± 0.1%, *P* ≤ 0.001; Figures 1(b) and 2(b)). Moreover, aUC patients had higher percentage of circulating IL-17A cells compared with iUC or healthy donors (11.7 ± 1.4% versus 3.4 ± 0.3% or 1.5 ± 0.1%, *P* ≤ 0.001). Inactive UC group had an increased frequency of IL-17A-expressing CD4^+^ T cell compared with iCD (*P* = 0.003).

### 3.3. Frequency of IL-4-Producing CD4^+^/CD14^−^ T Cells in IBD Patients

Anti-inflammatory/profibrogenic IL-4-expressing CD4^+^ T cell subset had an increased frequency in aCD patients when compared to iCD patients or healthy donors (4.3 ± 0.4% versus 3.3 ± 0.3% or 2.1 ± 0.2%, *P* ≤ 0.05; Figures 1(c) and 2(c)). Furthermore, aUC patients had higher percentage of circulating IL-4 cells compared with iUC or healthy donors (9.8 ± 0.8% versus 5.2 ± 0.4% or 2.1 ± 0.2%, *P* ≤ 0.001). It is noteworthy that inactive IBD patient group had higher IL-4-expressing CD4^+^ T cell percentage compared with healthy donors (*P* ≤ 0.028), where iUC patients had the highest levels of Th2 cells versus iCD patients (*P* = 0.003).

### 3.4. Peripheral IFN-*γ*-Producing CD4^+^/CD14^−^ T Cells in Patients with UC and CD

Pro-inflammatory/antifibrogenic IFN-*γ*-expressing CD4^+^ T cell subset was present in higher amounts in active and inactive IBD (CD and UC) patients compared with healthy donors (1.7 ± 0.2%; *P* = 0.001). aCD patients had lower frequency of IFN-*γ* ^+^ cells when compared to iCD patients (5.3 ± 0.4% versus 8.4 ± 0.6%, *P* ≤ 0.044; Figures 1(d) and 2(d)). In contrast, aUC patients had higher percentage of circulating IFN-*γ* cells compared with iUC (11.8 ± 0.9% versus 6.1 ± 0.3%, *P* ≤ 0.001). 

### 3.5. IL-10-Producing CD4^+^/CD14^−^ T Cells in IBD Patients

IL-10-expressing CD4^+^ T cell subset was present in higher amounts in active and inactive IBD (CD and UC) patients when compared to healthy donors (2.1 ± 0.2%, *P* ≤ 0.001). aCD patients had higher frequency of IL-10^+^ cells compared with iCD patients (6.9 ± 0.6% versus 4.5 ± 0.6%, *P* ≤ 0.001; Figures 1(e) and 2(e)). Also, aUC patients had higher percentage of circulating IL-10 cells versus iUC (11.5 ± 0.9% versus 6.5 ± 0.5%, *P* ≤ 0.001; Figures 1(e) and 2(e)). It is noteworthy that CD patients had lower IL-10-expressing CD4^+^ T cell percentage versus UC patients (*P* ≤ 0.02).

### 3.6. Frequency of Foxp3^+^ Regulatory T Cells in IBD Patients

Regarding lymphocytes that regulate the adaptive immune response and induce peripheral tolerance, CD4^+^/CD25^hi^/Foxp3^+^ cell subpopulation was quantified. The frequency of these cells in peripheral blood was higher in clinical active IBD patients compared with healthy donor group (3.9 ± 0.2%). aCD patients had higher frequency of Treg cells compared with iCD patients (10.4 ± 1.1% versus 4.7 ± 0.3%, *P* ≤ 0.001; Figures 1(f) and 2(f)). aUC patients had higher percentage of circulating Tregs cells versus iUC patients (11.1 ± 0.5% versus 6.5 ± 0.3%, *P* ≤ 0.001). Higher amount of Tregs was observed in iUC patients versus iCD patients and healthy donors (*P* = 0.001).

### 3.7. IDO Expression in Peripheral CD8*α* ^+^/CD16^+^ Cells in IBD Patients

A high expression of IDO was previously found in IBD and is thought to represent a mechanism for downregulation of proinflammatory response. Therefore, we determined the relative percentage of CD8*α* ^+^/CD16^+^/IDO^+^ cells based on percentage of CD8*α* ^+^/CD16^+^ double positive cells from total PBMCs (Figure 4(a)). Results showed an increased frequency of this subpopulation in aCD and aUC compared with iCD and iUC, respectively, or healthy controls (CD: 51.1 ± 2.2% versus 12.1 ± 1.0% or 30.9 ± 1.2%, *P* < 0.001; UC: 45.9 ± 2.6% versus 14.7 ± 0.9% or 30.9 ± 1.2%, *P* < 0.001; Figure 4(b)). It is remarkable that iCD and iUC patients had lower percentage of CD8*α* ^+^/CD16^+^/IDO^+^ cells compared with healthy donors (*P* < 0.001; Figure 4(b)).

### 3.8. IDO Expression in Circulating CD8*α* ^+^/CD56^+^ Cells in IBD Patients

**Figure 3:**
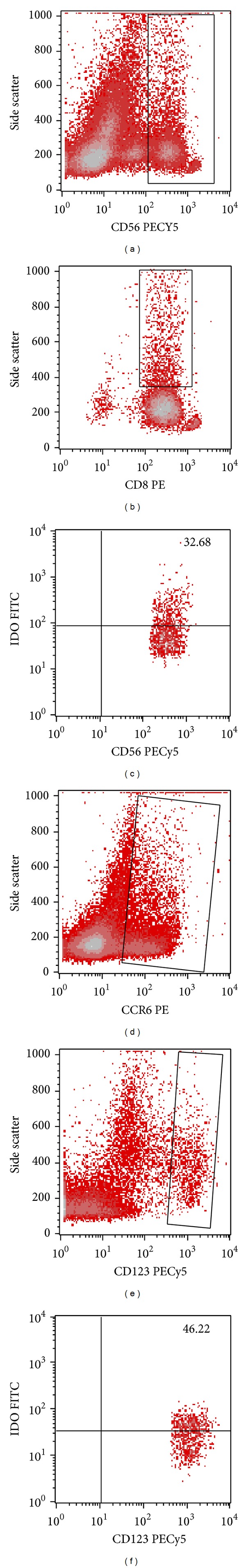
Representative density plots of IDO-expressing circulating cells in IBD (UC and CD) patients. (a) An electronic gate was made for CD56^+^ cells. (b) From the gate *A* CD56^+^/CD8*α* ^+^ large granular cells were determined. (c) From the latter CD56^+^/CD8*α* ^+^/IDO^+^ cells were defined. (d) An electronic gate was made for CCR6^+^ cells. (e) From the gate *D* CCR6^+^/CD123^+^ plasmacytoid dendritic cells were determined, and an electronic gate was made for double positive cells. (f) From the latter CCR6^+^/CD123^+^/IDO^+^ cells were defined. A total of 50,000–100,000 events were recorded for each sample before any gate setting and analysed with the CellQuest Pro software (BD Biosciences).

**Figure 4:**
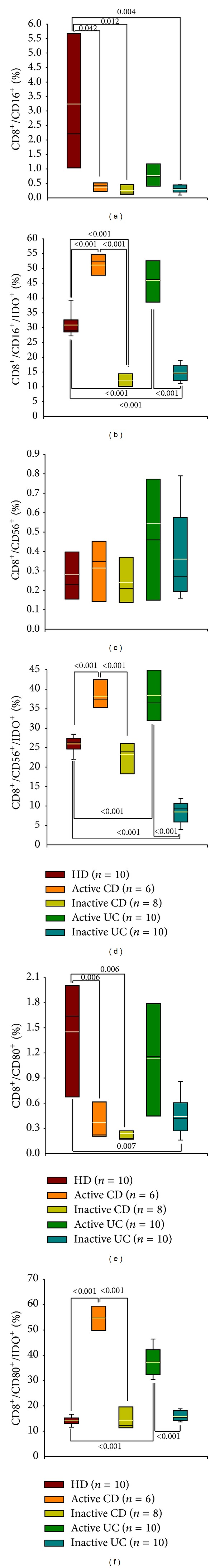
Percentage of IDO-expressing circulating cells in IBD (UC and CD) patients. Bar graphs show percentage of (a) CD16^+^/CD8*α* ^+^ large granular cells, (b) CD16^+^/CD8*α* ^+^/IDO^+^ cells, (c) CD56^+^/CD8*α* ^+^ large granular cells, (d) CD56^+^/CD8*α* ^+^/IDO^+^ cells, (e) CD80^+^/CD8*α* ^+^ large granular cells, and (f) CD80^+^/CD8*α* ^+^/IDO^+^ cells. Results are expressed as median (black line), 10th, 25th, 75th, and 90th percentiles. Yellow line represents the mean.

Relative percentage of CD8*α* ^+^/CD56^+^/IDO^+^ cells was obtained based on percentage of CD8*α* ^+^/CD56^+^ double positive cells from total PBMCs (Figures 3(a)–3(c) and 4(c)). Results showed an increased frequency of this subpopulation in aCD and aUC versus iCD and iUC, respectively, or healthy controls (CD: 38.2 ± 1.5% versus 23.2 ± 1.6% or 26.0 ± 0.7%, *P* < 0.001; UC: 38.4 ± 2.7% versus 8.5 ± 0.9% or 26.0 ± 0.7%, *P* < 0.001; Figure 4(d)). Of note, iUC patients had lower percentage of CD8*α* ^+^/CD56^+^/IDO^+^ cells compared with iCD or healthy donors (*P* < 0.001; Figure 4(d)).

### 3.9. IDO Expression in CD8*α* ^+^/CD80^+^ Cells in UC or CD Patients

Relative percentage of CD8*α* ^+^/CD80^+^/IDO^+^ cells was obtained based on percentage of CD8*α* ^+^/CD80^+^ double positive cells from total PBMCs (Figure 4(e)). Results showed an increased frequency of this subpopulation in aCD and aUC compared with iCD, iUC, or healthy controls (CD: 54.7 ± 2.5% versus 14.3 ± 1.6% or 14.1 ± 0.5%, *P* < 0.001; UC: 37.2 ± 1.8% versus 15.9 ± 0.7% or 14.1 ± 0.5%, *P* < 0.001; Figure 4(f)). CD8*α* ^+^/CD80^+^/IDO^+^ cell frequency in aCD was higher versus aUC (*P* < 0.001; Figure 4(f)).

### 3.10. IDO Expression in CD8*α* ^+^/CD123^hi^ Cells in IBD Patients

**Figure 5:**
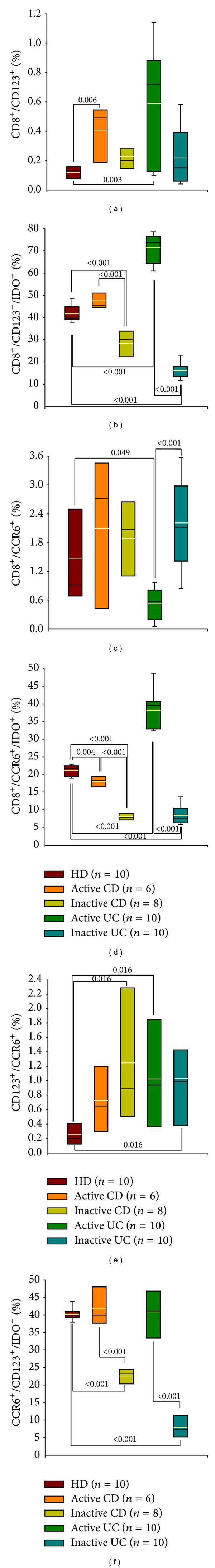
Percentage of IDO-expressing circulating cells in IBD (UC and CD) patients. Bar graphs show percentage of (a) CD123^+^/CD8*α* ^+^ large granular cells, (b) CD123^+^/CD8*α* ^+^/IDO^+^ cells, (c) CD8*α* ^+^/CCR6^+^ large granular cells, (d) CD8*α* ^+^/CCR6^+^/IDO^+^ cells, (e) CD123^+^/CCR6^+^ plasmacytoid dendritic cells, and (f) CD123^+^/CCR6^+^/IDO^+^ cells. Results are expressed as median (black line), 10th, 25th, 75th, and 90th percentiles. Yellow line represents the mean.

Relative percentage of CD8*α* ^+^/CD123^hi^/IDO^+^ cells was obtained based on percentage of CD8*α* ^+^/CD123^hi^ double positive cells from total PBMCs (Figure 5(a)). Results showed an increased frequency of this subpopulation in aUC versus iUC or healthy controls (71.3 ± 2.1% versus 16.2 ± 1.1% or 41.7 ± 1.3%, *P* < 0.001; Figure 5(b)). Remarkable is the low frequency of CD8*α* ^+^/CD123^hi^/IDO^+^ subset in iUC compared with aUC, iCD patients, and healthy donors (*P* ≤ 0.001; Figure 5(b)). iCD patients had lower levels of CD8*α* ^+^/CD123^hi^/IDO^+^ cells versus aCD and healthy donors (28.3 ± 2.6% versus 47.5 ± 2.2% or 41.7 ± 1.3%, *P* ≤ 0.001; Figure 5(b)). 

### 3.11. IDO Expression in Circulating CD8*α* ^+^/CCR6^+^ Cells in Patients with UC or CD

Relative percentage of CD8*α* ^+^/CCR6^+^/IDO^+^ cells was obtained based on percentage of CD8*α* ^+^/CCR6^+^ double positive cells from total PBMCs (Figure 5(c)). Results showed an increased frequency of this subpopulation in aUC compared with iUC patients or healthy controls (38.2 ± 1.8% versus 8.3 ± 0.9% or 21.2 ± 0.5%, *P* < 0.001; Figure 5(d)). Outstanding is the low frequency of CD8*α* ^+^/CCR6^+^/IDO^+^ cells in aCD (17.9 ± 0.7%), iCD (8.0 ± 0.4%), and iUC compared with aUC and healthy donors (*P* < 0.04; Figure 5(d)).

### 3.12. Peripheral CD123^hi^/CCR6^+^/IDO^+^ Plasmacytoid Dendritic Cells

Recently, a subpopulation of plasmacytoid dendritic cells derived from monocytes expressing CD123^hi^/CCR6^+^/IDO^+^ that are responsible for mediating the suppression of effector T cells it has been described. Relative percentage of CD123^hi^/CCR6^+^/IDO^+^ cells was obtained based on percentage of CD123^hi^/CCR6^+^ double positive cells from total PBMCs (Figures 3(d)–3(f) and 5(e)). The frequency of these cells was akin among aCD and aUC groups when compared to healthy donors (Figure 5(f)). It is remarkable that inactive IBD had statistically significant lower levels compared with active disease or healthy donors (CD: 22.6 ± 1.0% versus 41.7 ± 2.2% or 40.2 ± 0.5%, *P* < 0.001; UC: 7.9 ± 1.1% versus 40.8 ± 2.4% or 40.2 ± 0.5%, *P* < 0.001; Figure 5(f)). However, the lowest CD123^hi^/CCR6^+^/IDO^+^ cell frequency was determined in iUC patients (*P* < 0.001; Figure 5(f)).

### 3.13. Percentage of IL-17A-Expressing Cells on Tissue from IBD Patients

**Figure 6:**
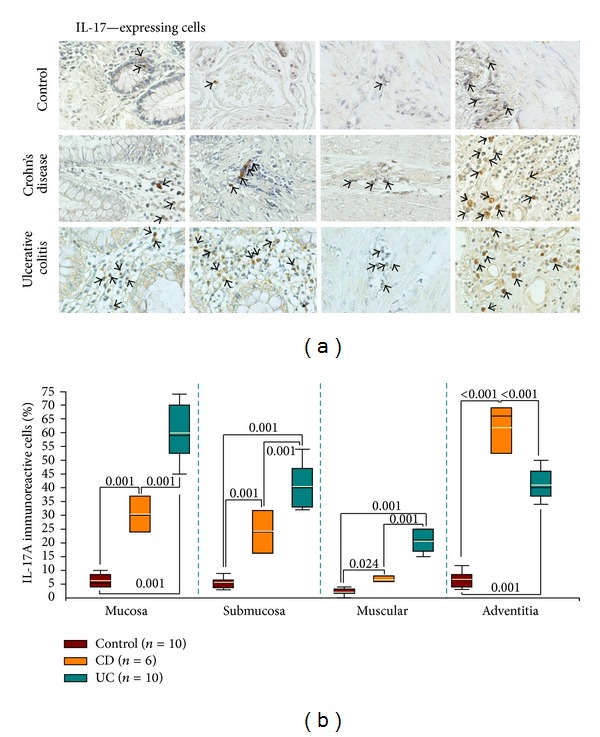
IL-17A-expressing cells in colonic mucosa from IBD (UC and CD) patients. (a) Representative immunoperoxidase of IL-17A expression in non-inflamed colonic tissue (upper panel), in tissue from an active CD patient (middle panel), and in colonic mucosa from an active UC patient (lower panel). From the left to the right photomicrographs represent mucosa, submucosa, muscular, and adventitia. Arrows depict immunoreactive positive cells (X320). (b) Bars indicate immunohistochemistry analysis of IL-17A-producing cells in non-inflamed colonic tissues, in tissues from active CD patients, and in colonic mucosa from active UC patients. Results are expressed as median (black line), 10th, 25th, 75th, and 90th percentiles. Yellow line represents the mean.

In order to determine *in situ* IL-17A protein expression from aUC and aCD intestinal biopsies, tissues were immunostained and compared with non-inflamed tissues. The percentage of IL-17A immunoreactive cells was higher in UC and CD patients compared with controls (*P* < 0.02; Figures 6(a) and 6(b)). Furthermore, IL-17A-producing cells were conspicuously increased in UC patients compared to CD patients; positive cells were localized mainly in mucosa, lamina propria, submucosa, muscular, and perivascular inflammatory infiltrates but not in goblet cells, crypt lumen, or crypt blanching. IL-17A^+^ cells were lower in adventitia from UC versus CD patients (*P* < 0.001).

### 3.14. Percentage of CD123/IDO-Expressing Cells on Tissue from UC or CD Patients

**Figure 7:**
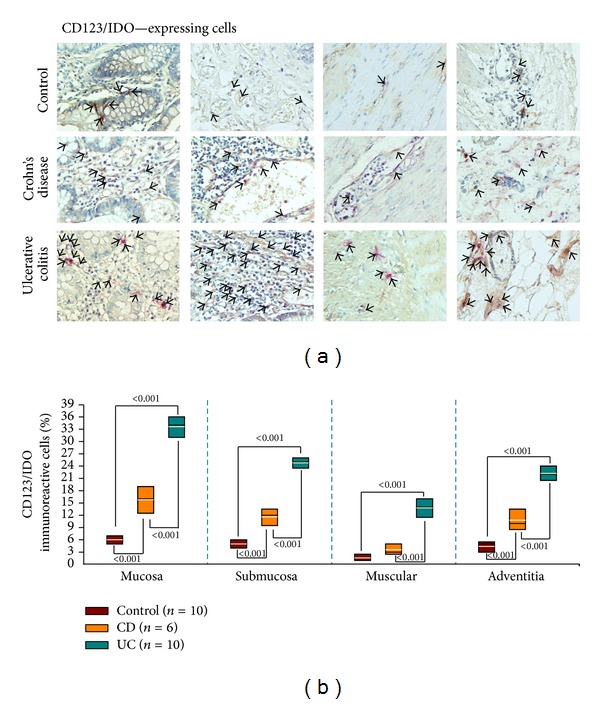
CD123^+^/IDO^+^ cells in colonic mucosa from IBD (UC and CD) patients. (a) Representative immunoperoxidase of CD123/IDO expression in non-inflamed colonic tissue (upper panel), in tissue from an active CD patient (middle panel), and in colonic mucosa from an active UC patient (lower panel). From the left to the right photomicrographs represent mucosa, submucosa, muscular, and adventitia. Arrows depict CD123 (in brown)/IDO (in pink) immunoreactive positive cells (X320). (b) Bars indicate immunohistochemistry analysis of IDO-producing cells in non-inflamed colonic tissues, in tissues from active CD patients, and in colonic mucosa from active UC patients. Results are expressed as median (black line), 10th, 25th, 75th, and 90th percentiles. Yellow line represents the mean.

IDO is primarily expressed in CD123^+^ dendritic cells infiltrating areas of the inflamed lesions. This subpopulation has been shown to exert potent inhibitory effects on T cell-proliferation and depletion of tryptophan. Thus, overexpression of IDO in CD123^+^ dendritic cells was determined on mucosa, submucosa, muscular, and adventitia from aUC patients compared with aCD patients and non-inflamed control tissue (*P* ≤ 0.001; Figures 7(a) and 7(b)). In turn, aCD patients showed a statistically significant increase in percentage of CD123^+^/IDO^+^ double positive cells when compared with control tissue.

### 3.15. Percentage of CD25/Foxp3-Expressing Cells on Tissue from UC or CD Patients

**Figure 8:**
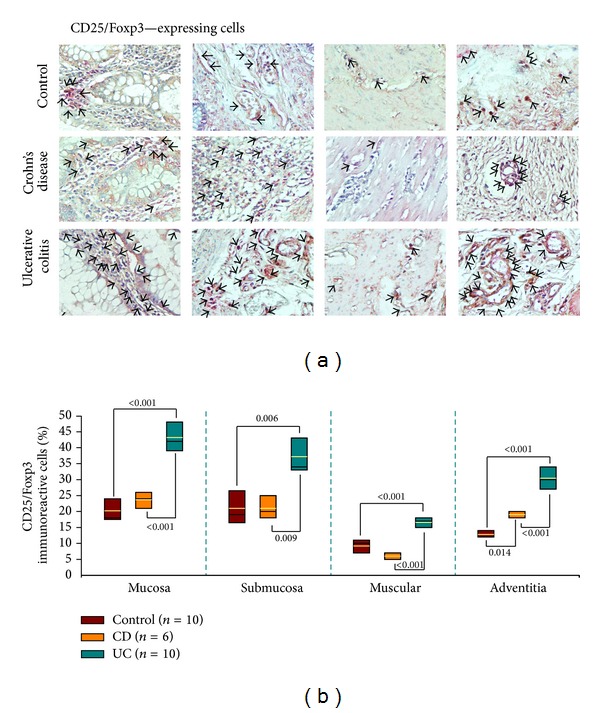
CD25^+^/Foxp3^+^ cells in colonic mucosa from IBD (UC and CD) patients. (a) Representative immunoperoxidase of CD25/Foxp3 expression in non-inflamed colonic tissue (upper panel), in tissue from an active CD patient (middle panel), and in colonic mucosa from an active UC patient (lower panel). From the left to the right photomicrographs represent mucosa, submucosa, muscular, and adventitia. Arrows depict CD25 (in brown)/Foxp3 (in pink) immunoreactive positive cells (X320). (b) Bars indicate immunohistochemistry analysis of CD25^+^/Foxp3^+^ cells in non-inflamed colonic tissues, in tissues from active CD patients, and in colonic mucosa from active UC patients. Results are expressed as median (black line), 10th, 25th, 75th, and 90th percentiles. Yellow line represents the mean.

Foxp3 regulatory T cells are critical for controlling inflammation in the gastrointestinal tract. For this reason, CD25^+^/Foxp3^+^ Treg cells were determined in tissue from IBD patients. The frequency of Tregs on mucosa, submucosa, muscular, and adventitia from aUC patients was higher compared with aCD patients and non-inflamed control tissue (*P* ≤ 0.006; Figures 8(a) and 8(b)). No differences were found in CD25^+^/Foxp3^+^ immunoreactive cell percentage on mucosa, submucosa, and muscular layers of aCD patients when compared with non-inflamed control tissue.

## 4. Discussion

IBD is a chronic inflammatory state of the gastrointestinal tract of unknown aetiology. Classically, tissue injury in IBD is thought to be primarily mediated by Th1 cells in CD or Th2 in UC [21], as we observed in our Mexican Mestizo patients. Recently, Th17 cells have been introduced as a third and new lineage of Th cells that seem to affect both innate and adaptive immune responses by the release of proinflammatory cytokines and their involvement in autoimmune disorders [22]. The concept of breaking of tolerance is fundamental to the development of autoimmunity. The first and perhaps most vital stage of tolerance induction to self-antigens occurs in the thymus during T cell development. Because proteins with tissue-restricted or peripheral expression are traditionally thought to be unavailable for presentation in the thymus, it has been proposed that tolerance to such proteins can only be achieved through mechanisms of peripheral tolerance [23]. The role of IDO and the specific IDO-expressing cells in normal and disease conditions has not been yet fully characterised. Significant interest has been generated, however, in manipulating IDO expression in transplantation, HIV, tumour resistance, and autoimmunity [24–26]. The expression of IDO by different cell types, as well as the capacity of IDO competent DCregs to induce Treg cells through high levels of IDO activity, could have broader immunological significance in tolerance and immunoregulation; for example, it has been suggested that defects in the immunoregulatory mechanism initiated by IDO are involved in the development of autoimmune conditions, such as multiple sclerosis, systemic lupus erythematosus, rheumatoid arthritis, and autoimmune diabetes [27–29]. In this vein, our results showed an accumulation of IDO^+^/CD123^+^ plasmacytoid dendritic cells in colonic biopsies of IBD patients. The findings are consistent with those of Wolf et al.'s and Zhou et al.'s studies which showed increased expression of IDO mRNA and protein in IBD Austrian and Chinese patients, respectively. They suggest that high local IDO expression may represent an anti-inflammatory mechanism tempting to counterbalance the tissue-damaging effects of activated T cells infiltrating the colonic mucosa in IBD [30, 31]. On the other hand, our results also showed that in aUC and aCD patients there was a conspicuously increase in the number of CD8*α* ^+^/CD16^+^/IDO^+^, CD8*α* ^+^/CD56^+^/IDO^+^, CD8*α* ^+^/CD80^+^/IDO^+^, and CD8*α* ^+^/CD123^+^/IDO^+^ large granular cells when percentages were compared to healthy controls. Meanwhile, in iCD and iUC patients, there was a marked decrease of IDO-expressing cell frequency when percentages were compared to active IBD or healthy donors. These IDO-expressing subsets have not been previously described, and we suggest that it could be a plasmacytoid DC, according to blood DC subsets described by Dzionek et al. Indeed one of these pDC subsets displays the expression of CD123^hi^ and pre-TCR *α*-chain [32]. Generally, already small amounts of IDO^+^ DCs are sufficient to promote immune suppression, directly or by means of bystander suppression [10]. In mice, as few as 3% of CD8*α* ^+^/IDO^+^ DC has been reported to suppress the stimulatory effects of significantly higher amounts of CD40-activated CD8*α* ^−^ DC *in vivo* [33, 34]. CD123^+^/IDO^+^ cells pDCs constitute only 0.2–0.8% of peripheral blood cells and represent a unique rather plastic, versatile, and important immune cell population capable of producing over 95% of IFN-I synthesized by peripheral blood mononuclear cells in response to viruses as well as nucleic acid-containing complexes from the host. In addition to IFN-I, human pDCs produce proinflammatory cytokines such as TNF-*α* and IL-6 in response to TLR activation [35]. Manlapat et al. have recently shown that the secretion of INF-*α* is indispensable for high-level expression of IDO after B7·1/B7·2 ligation to CTLA-4 [32, 36, 37]. Human pDCs possess an exclusive TLR repertoire, TLR7 and TLR9, express several unique surface receptors, but do not produce IL-12p70 or IL-10. CpG-activated human pDCs upregulate B7 ligands, HLA-DR, and IFN-I production, through TLR9. IFN-I upregulates IDO expression in CpG-activated human pDCs, contributing to regulatory T cell generation (CD4^+^/CD25^+^/Foxp3^+^) from CD4^+^/CD25^−^ T cells with potent suppressor cell function [38]. IDO-expressing and Treg peripheral cells increased percentage in active IBD patients may be a compensatory mechanism for functional induction of tolerance in active disease, due to the increase in absolute number of circulating IL-17A-, IL-4-, and IFN-*γ*-expressing cell percentage. In this case, IDO expression could merely be an indicator of the strength of an immune response or Th17, Th1, and Th2-related inflammation and act as a counter-regulatory response [39, 40].

On the other hand, the scant IDO-peripheral cells frequency in inactive IBD patients is consistent with the findings of our group in rheumatoid arthritis and systemic lupus erythematosus patients and in human primary biliary cirrhosis [27, 41], and it might be related to the recruitment of blood derived-DC at the tissue in order to induce tolerance [42]. This could be true for CD123/IDO and CD25/Foxp3 cells as we determined in biopsies from clinical active patients. These data suggest that increased IDO expression in the gut may be used as a prognostic factor or diagnostic biomarker. 

In summary, tolerogenic mechanisms in aUC patients might be based on the increase of IDO-expressing cells CD8*α* ^+^/CD16^+^/IDO^+^, CD8*α* ^+^/CD56^+^/IDO^+^, CD8*α* ^+^/CD80^+^/IDO^+^, CD8*α* ^+^/CD123^+^/IDO^+^, CD8*α* ^+^/CCR6^+^/IDO^+^, IL-10-producing CD4 T cells, and Tregs in a proportional rate of increment of Th17, Th2, and Th1. These IDO^+^ cell subsets might actively participate as a compensatory mechanism to develop peripheral tolerance inducing the polarization of CD4^+^ T cells to Treg cells and suppressing Th17, Th2, and Th1 differentiation, albeit functional assessments of IDO-expressing cells are required. In contrast, in aCD patients tolerogenic cells are at lower levels when compared with aUC patients and healthy donors. Our results shed further light into the preponderant role of IDO in autoimmune disorders and certainly deserve to be studied in depth in order to evaluate the clinical relevance of these findings.

## Figures and Tables

**Table 1:** Demographic and clinical characteristics of Crohn's disease and ulcerative colitis patients.

Variable	Healthy donors (*n* = 10)	Active CD patients (*n* = 6)	Inactive CD patients (*n* = 8)	Active UC patients (*n* = 10)	Inactive UC patients (*n* = 10)
Age, years					
Mean ± SD	40.3 ± 6.7	45.0 ± 18.4	40.0 ± 9.3	44.7 ± 14.9	41.5 ± 14.9
Median	40.0	44.0	42.0	46.0	43.0
Range	29–51	22–67	25–52	20–69	20–70
Sex, female/male	4/6	1/5	1/7	3/7	5/5
Disease duration, years					
<3		0%	25%	0%	10%
>3		100%	75%	100%	90%
Treatment					
Mesalazine		5/6	0/8	10/10	10/10
Azathioprine		1/6	2/8	7/10	3/10
Prednisone		4/6	0/8	9/10	1/10
Mercaptopurine		1/6	1/8	0/10	0/10
Disease extension					
Distal colitis		0/6	8/8	3/10	7/10
Pancolitis		6/6	0/8	7/10	3/10
Extraintestinal manifestations					
Absent		6/6	8/8	8/10	7/10
Present		0/6	0/8	2/10	3/10

CD: Crohn's disease patient group; UC: ulcerative colitis patient group.

**Table 2:** Laboratory variables of Crohn's disease and ulcerative colitis patients.

Variable	Healthy donors (*n* = 10)	Active CD patients (*n* = 6)	Inactive CD patients (*n* = 8)	Active UC patients (*n* = 10)	Inactive UC patients (*n* = 10)
ESR, mm Hg					
Mean ± SD		19.6 ± 10.9	5.9 ± 2.8^a^	28.4 ± 14.4	6.5 ± 4.3^b^
Median		18.0	6.0	27.0	6.0
Range		7–36	2–11	15–57	1–71
CRP, mg/dL					
Mean ± SD		2.8 ± 3.2	0.34 ± 0.22^c^	0.82 ± 0.71	0.30 ± 0.16
Median		0.97	0.28	0.71	0.30
Range		0.1–6.5	0.06–0.80	0.1–1.9	0.02–2.98
Haemoglobin, g/dL					
Mean ± SD		13.2 ± 1.4	15.7 ± 1.6^d^	12.9 ± 3.1	14.3 ± 2.6
Median		12.7	15.9	12.2	15.1
Range		11.5–15.1	13.1–18.1	9.8–18.2	8.9–17.0
Platelets, cells/L					
Mean ± SD		255.2 ± 72.9	216.7 ± 33.5	318.7 ± 128.9	286.8 ± 113.3
Median		288.0	214	292.0	227.0
Range		161–322	161–258	146–570	116–518
Leucocytes, ×10^9^ cells/L					
Mean ± SD		5.9 ± 1.6	5.7 ± 1.1	7.1 ± 3.0	6.2 ± 2.1
Median		6.0	5.6	6.5	5.7
Range		4.4–8.4	4.2–8.2	2.4–12.6	3.5–10.3
Lymphocytes, %					
Mean ± SD		19.2 ± 11.3	26.9 ± 9.3	29.0 ± 8.9	28.0 ± 8.0
Median		18.0	27.7	29.0	28.3
Range		8.3–37.8	14.2–41.0	15.0–41.5	18.0–45.2
Monocytes, %					
Mean ± SD		7.7 ± 4.2	7.7 ± 2.3	7.7 ± 3.2	6.7 ± 2.1
Median		6.4	7.0	8.1	6.4
Range		4.3–15.0	4.5–11.3	2.0–14.6	4.0–11.2
Polymorphonuclear cells, %					
Mean ± SD		68.3 ± 8.6	64.1 ± 10.5	58.6 ± 13.7	60.5 ± 13.9
Median		70.5	63.7	61.4	62.0
Range		53.7–75.6	50.0–77.5	34.0–75.0	23.0–77.0

CD: Crohn's disease patient group; UC: ulcerative colitis patient group.

^a^Active CD versus inactive CD; *P* = 0.003.

^b^Active UC versus inactive UC; *P* < 0.001.

^c^Active CD versus inactive CD; *P* = 0.035.

^d^Active CD versus inactive CD; *P* = 0.010.
